# Supplementary material for: Are carbon emissions trading and green financial instruments synergistic? -Comprehensive quantitative research based on content analysis
Source: PLoS One. 2024 Mar 7;19(3):e0298601. doi: 10.1371/journal.pone.0298601 (PMC10919601; doi:10.1371/journal.pone.0298601)
Supplement: S1 Data — (ZIP) [file pone.0298601.s001.zip › Data/地级市Z_F工作报告.zip!/╔╜╢1⁄2/╟α╡║╩╨/└┤╫╘╣1⁄2╓┌║┼í╛┬φ┐╦╩2╛▌═°í┐_2007.docx]

各位代表：

　　现在，我代表市人民政府向大会作工作报告，请予审议，并请各位政协委员和其他列席人员提出意见。

　　一、2006年工作回顾

　　2006年，是“十一五”规划开局之年。在党中央、国务院，省委、省政府和市委的领导下，我们紧紧依靠全市人民，坚持以邓小平理论和“三个代表”重要思想为指导，全面落实科学发展观，努力构建社会主义和谐社会，积极推进经济建设、政治建设、文化建设和社会建设，较好地完成了市十三届人大四次会议确定的目标任务，实现了“十一五”的良好开局。

　　初步预计，全市生产总值超过3100亿元，增长15%以上；地方财政一般预算收入225.8亿元，增长28%。城市居民人均可支配收入15328元，农民人均纯收入6546元，分别增长18.6%和12.7%。城镇登记失业率2.96%，居民消费价格指数上升0.9％。人口自然增长率为3％。。

　　（一）加快推进结构调整，经济保持平稳较快发展。一年来，我们注重经济发展的稳定性和协调性，结构调整和产业升级有新进展。106个重点项目顺利推进。南车动车组、前湾港南港区等项目开工建设，海洋石油工程、招商局码头、北汽福田挖掘机、泰科电子等项目相继建成。以信息化带动工业化，完成了11个传统工业信息化改造示范项目。引进各类金融机构7家，金融业实现税收增长33.4%。港口货物、集装箱吞吐量分别达到2.24亿吨和770万标箱。旅游业总收入增长25%左右。极地海洋世界、国际会展中心二期等项目建成。中国国际消费电子博览会、青岛国际啤酒节等重要展会的影响进一步扩大。三产占全市生产总值的比重上升，实现税收占地方税收的61.9%。新增中国名牌产品10个，再次荣获“中国品牌之都”。

　　在结构调整中，我们认真落实国家宏观调控政策。依法加强土地管理，集约利用土地，坚持有保有压，优化信贷投向，严格固定资产投资管理，全面清理新开工项目。规模以上固定资产投资增长20％左右，比上年回落25个百分点。认真执行国家房地产调控政策，初步建立起多层次住房供应体系，住房供应结构开始调整，房价增长过快的势头得到抑制。

　　（二）提高自主创新能力，增长方式加快转变。一年来，我们制定了中长期科学技术发展规划，实施了推进创新型城市建设的意见。引进中科院生物能源与过程研究所等6家国家级科研机构，海洋科学与技术国家实验室建设正式启动，国家级企业技术中心达到11家。鼓励企业抓好无线射频技术、太阳能光热电、智能交通等关键技术攻关。海尔、海信成为国家创新型试点企业。高新区顺利扩区。全市发明专利授权量增长19%。高新技术产业产值占规模以上工业总产值比重达到44%。

　　循环经济和清洁能源建设成效显著。奥林匹克帆船中心太阳能利用、风能照明、海水源热泵等项目竣工使用，海水淡化、铬渣利用、白泥利用、农用沼气等项目加快推进。节能降耗和环境保护工作不断加强。全面落实节能减排目标，对110家重点用能企业实行严格管理和考核制度。工业固体废弃物综合利用率达97％。新建成节能建筑面积592万平方米。麦岛污水处理厂扩建和墨水河综合治理二期工程竣工。市区空气质量优良率达到91%。建成国家环保模范城市群。

　　（三）加强城市规划建设管理，城市功能不断完善。一年来，我们完成了滨海公路控制性详规、海岸带保护利用及控制性规划、海洋功能区划的编制，新一轮城市总体规划和土地利用总体规划修编有序进行。胶济铁路电气化改造竣工，滨海公路北段全线贯通，机场扩建、火车站改造、即平高速等项目进度加快。胶州湾海底隧道、海湾大桥主体工程开工建设。杭州支路—鞍山路快速路主线通车，长沙路打通工程顺利实施。城市绿化和环境综合整治力度加大，清除违法建筑132万平方米。顺利通过国家卫生城市复审。

　　（四）扎实推进社会主义新农村建设，农村经济社会发展势头良好。一年来，现代农业建设进程加快，建成100万亩高产优质高效粮食示范区，实施了良种繁育和国家级畜产品生产基地建设，农业新技术推广应用工作深入推进。农业产业化经营规模扩大，新增省级以上龙头企业11家。平度、莱西的工业化取得新成效，五市全部进入国家综合实力百强县。

　　全面落实支农惠农政策，市财政支农资金达到13.7亿元，比上年增长44.2%。发放粮食直补等三项补贴和农资综合直补1.3亿元。完成了967个村通自来水工程，行政村柏油路通达率提高到89%。实施了小型病险水库除险加固、节水灌溉工程。农村义务教育经费保障机制基本建立，市本级财政新增教育经费1.2亿元，提前一年免除55万农村义务教育阶段学生的杂费。农村合作医疗财政补助标准、农村最低生活保障标准分别提高1倍和31.7%，新型农村合作医疗参合率达到96%，被征地农民社会基本养老保险参保率超过90%。完成了90所镇卫生院标准化和3119个村卫生室规范化达标建设，扩建改造了64个镇综合文化站和1200个村文化活动室。全面完成1000户农村贫困残疾人安居工程。加强了对库区移民村的帮扶。稳步推进农村综合改革，加大了对困难镇村的财政转移支付力度。

　　（五）深化改革扩大开放，体制机制更加富有活力。一年来，积极推进和深化重点领域改革。《青岛市企业国有资产监督管理暂行办法》颁布实施。加强了国有重点企业经营风险防范控制。青岛啤酒等10家上市公司股权分置改革、黄海橡胶等6家企业调整重组全面完成，康大集团、青岛软控、青岛金王等公司成功上市。民营经济加快发展，增加值占全市比重达到50%，吸纳就业占全市新增就业的70%。政府投资体制、政府收支分类、出租车管理运营体制、资源性产品价格等改革稳步推进。

　　对外开放取得新进展。利用外资质量有新提高，引进精密机电、风力发电等装备制造业和海水淡化等循环经济项目，渣打银行和普华永道、毕马威等会计师事务所落户我市，全年实际利用外资36.5亿美元。外贸增长方式加快转变，进出口总额365.6亿美元，增长20%；机电产品和高新技术产品出口，分别增长30.8%和51.1%。青岛西海岸出口加工区获国家批准。海尔—鲁巴经济区成为我国首个境外经贸合作区。商务环境进一步改善，荣获世界银行中国投资环境“金牌城市”称号。外事、侨务、对台、口岸、对口支援等工作不断加强。

　　（六）更加关心民生，居民生活质量持续改善。一年来，我们坚持以人为本、以民生为重，认真解决关系人民群众切身利益的问题。市政府确定重点办好的10件实事全部完成。城乡居民收入进一步增加。企业最低工资标准提高15.1%，企业退休人员养老金人均增长23.5%。各级财政安排促进就业资金3.8亿元，全年实现城乡就业28万人。社会保障覆盖范围进一步扩大，企业养老保险、医疗保险新增参保职工分别为20万人，工伤、生育保险参保人数首次超过100万人。城市最低生活保障人员基本实现应保尽保。慈善、福利事业加快发展。对80岁以上老人体检实施补助，新增机构养老床位1600张。经济适用房竣工83万平方米。解决了3377户廉租住房。新增供热面积163万平方米。完成10.3万户自来水“一户一表”改造。建成并投入使用公交停车场5处，公交车占路停车状况有较大改善。

　　“平安青岛”建设成效明显。社会治安综合治理得到加强，刑事案件、群体性上访、安全生产事故起数和死亡人数分别下降2%、22.6%、22.7%和9.1%。深入开展矛盾纠纷排查调处，行政、司法和人民调解相衔接的“大调解”工作机制基本建立，调处化解群众矛盾的能力进一步增强。公共安全应急体系不断完善，应急管理体制初步形成。“食品药品放心工程”深入实施，市场秩序得到进一步整顿和规范。

　　（七）推动经济社会协调发展，社会事业取得新进步。一年来，市财政对医疗、教育、文化事业的支出分别增长27.1%、31.1%和17.6%。优化教育资源配置，市内四区初中管理体制调整顺利完成，教育质量有所提高，职业教育和高等教育等全面发展。市立医院东部院区、市传染病医院病房投入使用，完成了60个社区卫生服务机构标准化建设。改扩建了67处社区文化中心。市体育运动学校迁建工程竣工。青岛大剧院、市体育馆等重点设施加快建设。成功举办了全国第八届小提琴演奏比赛、国际音乐大师班和2006青岛音乐节。青岛文化街、达尼画家村被命名为“国家文化产业示范基地”。在第15届亚运会和第21届省运会上，青岛籍运动员和青岛体育代表团取得了历史最好成绩。出台并实施了加强和谐社区建设的意见。积极践行社会主义荣辱观，深入开展“迎奥运、讲文明、树新风”等活动。完善人口计生新机制建设，推进优生工程，低生育水平保持稳定。双拥和民兵预备役工作进一步加强，各区市人武部达标建设顺利推进。妇女儿童、老龄和残疾人事业实现新发展，广播电视、新闻出版、民族宗教、档案史志、气象、地震、红十字、人防、仲裁等工作取得新成绩。

　　（八）加强政府自身改革与建设，政府行政能力进一步提高。一年来，我们坚持为民、务实、清廉的要求，政府科学、民主、依法行政的能力不断增强。自觉接受人大及其常委会的法律监督、工作监督和政协的民主监督，加强同各民主党派、工商联、无党派人士和各人民团体的联系，虚心听取意见建议。认真办理人大代表议案、建议和政协提案、建议案，筹建国医堂、加强农科院建设两个议案进展顺利。认真学习《监督法》，深入贯彻《行政许可法》，做好实施《公务员法》的各项准备工作，不断完善目标管理绩效考核体系。创建高绩效机关、做人民满意公务员工作取得新的成效。对经济社会发展和涉及民生的重要决策，及时召开听证会或进行社会公示。规范财政专项资金的使用管理，将社保基金、各类公益金、专项资金纳入重点监管。电子政务建设成效显著，初步实现了“一网式”办公和“一站式”公共服务。切实纠正损害群众利益的不正之风，全面强化建设工程招投标、政府采购、重大项目前期介入、行政审批等方面的监督制度，严厉查处商业贿赂案件，教育、制度、监督并重的惩治和预防腐败体系进一步完善。

　　去年，经过全市人民的共同努力，我们高标准、高质量、高效率地完成了青岛奥林匹克帆船中心主体工程，成功举行了规模大、规格高、影响广的2006青岛国际帆船赛，城市运行、赛事组织、服务保障、安全保卫、迎奥宣传得到全面检验，赢得国内外广泛赞誉，交出了通向2008的第一份合格答卷。

　　各位代表！过去一年的成就来之不易。青岛发展取得的每一个成绩，政府工作取得的每一点进步，都凝聚着全市人民的智慧和力量，得益于全市人民的理解和支持。在此，我谨代表市人民政府，向为青岛发展做出突出贡献的全市人民，向历届市领导班子的各位老同志致以崇高的敬意！向各位人大代表、政协委员和社会各界人士，向中央、省驻青单位，人民解放军和武警驻青部队，向所有来青的建设者和劳动者，以及关心支持青岛发展的海内外朋友们，表示衷心的感谢！

　　在肯定成绩的同时，我们也清醒地看到，经济社会发展还面临一些矛盾和困难，政府工作还存在一些问题和不足。主要是：结构调整任务依然艰巨，服务业发展滞后，自主创新能力有待提高，能源资源的利用水平相对较低，依法利用和管理土地需要加大力度；国有企业经营风险依然较大，国有资产监管亟待加强；公共服务水平和社会保障能力还不够高，住房、医疗、教育等问题解决得还不够好；农村生产生活条件需要进一步改善，农民增收难度较大。政府自身建设存在薄弱环节，一些制度规定执行不严、落实不够、监督不力。对上述问题，我们将采取有力措施，认真加以解决。

　　二、2007年工作总体安排

　　2007年，是全面完成本届政府施政目标的最后一年，也是实施“十一五”规划的关键一年。政府工作的总体要求是：以邓小平理论和“三个代表”重要思想为指导，认真贯彻党的十六大及十六大以来历次中央全会精神，全面落实科学发展观，加快构建社会主义和谐社会，按照“三个走在前面”的要求，站在高起点、瞄准大目标、实现新跨越，继续贯彻宏观调控政策，着力调整经济结构和转变增长方式，着力加强资源节约和环境保护，着力推进改革开放和自主创新，着力促进社会发展和解决民生问题，努力实现又好又快发展。

　　经济社会发展的主要预期目标是：全市生产总值增长13%，地方财政一般预算收入增长17%，城市居民人均可支配收入和农民人均纯收入分别增长10%，城镇登记失业率控制在4%以内，居民消费价格指数增幅控制在3%以内。主要约束目标是：万元生产总值综合能耗下降4.8%左右，化学需氧量、二氧化硫排放量均削减6%，人口自然增长率控制在4.6％。以内。

　　在工作指导上，重点把握好以下几个方面：

　　──切实把科学发展观落到实处，保持又好又快发展势头。深化对科学发展观的认识，完善科学发展的决策机制和政策措施，坚持好中求快，更加注重提高质量和效益，更加注重可持续协调发展，推动经济社会转入科学发展的轨道。

　　──坚持以人为本，积极营造共建共享和谐的生动局面。认真落实发展为了人民、发展依靠人民、发展成果由人民共享、促进人的全面发展的要求，更加关注民生、体察民情、尊重民意，充分考虑人民群众的利益诉求，使广大群众得到更多实惠。

　　──全面深化改革扩大开放，建立充满活力的体制机制。推进重点领域的改革，加快解决制约发展的体制性矛盾，为科学发展提供内生动力，为社会和谐奠定制度基础。深入实施互利共赢的开放战略，在更高层次、更高水平上扩大开放。

　　──加快推进施政创新，提高科学、民主、依法行政的能力。加快推进政府职能转变，全面履行政府责任，切实加强作风和廉政建设，提高政府的决策水平和公信力，努力建设法治政府、责任政府、廉洁政府、高效政府。

　　各位代表！全面完成2007年的各项任务，圆满实现本届政府的施政目标，是历史赋予我们的责任，是人民寄予我们的期望。我们要努力做到思想更加统一，精力更加集中，工作更加用心，作风更加扎实，不断开创科学发展、和谐发展的新局面。

　　三、坚持科学发展，加快提升经济发展质量

　　以先进制造业、高新技术产业、现代服务业为重点，以自主创新、节能降耗、环境保护和集约用地为突破口，推动城乡统筹协调发展，促进结构调整和增长方式转变。

　　（一）促进经济结构优化升级

　　以产业集群建设推动新型工业化。坚持一手抓核心项目的引进和建设，一手抓配套项目的引导和协作，大力发展装备制造业，形成一批生产企业集群，建成一批加工制造园区。加快推进大炼油、海西湾造修船基地、上汽通用五菱整车及发动机等重点产业项目建设。争取国家尽快核准前湾港区新建码头等重点项目。选择一批有条件的传统行业和产品，加大投入和技术改造力度，推动转型升级。加快发展信息技术、生物医药、新材料等高新技术产业，进一步提高高新技术产业产值占规模以上工业总产值的比重。壮大提升品牌经济，重点扶持自主品牌发展，促进“老字号”品牌振兴，争创更多的中国名牌。

　　推动现代服务业十大重点领域加快发展。完善促进金融业发展的政策措施，积极引进培育各类金融机构，不断创新金融产品，提高服务质量，改善金融环境。抓好旅游大项目建设，科学合理地保护、开发旅游资源，提高旅游业的营销质量和服务水平，争创首批中国最佳旅游城市。积极推进中远物流、空港物流等物流园区建设，加快发展第三方物流。大力培育工业设计、咨询服务、软件等创意产业。加强三级商业中心建设，繁荣消费市场。精心组织好中国国际消费电子博览会、青岛国际啤酒节、中日韩三国旅游部长会议等重大活动。

　　做强做大民营经济。引导民营企业参与产业集群配套、融入县域工业化、发展现代服务业。推进“中小企业成长工程”，重点扶持200家成长型中小企业。出台《中小企业法》实施办法，制订中小企业信用担保体系建设意见，构建融资、创业、信息、技术、培训五大服务平台。发挥行业协会、民间商会的作用，健全投诉受理和监督机制，营造公平的市场环境。

　　认真落实国家宏观调控政策。严把土地、信贷闸门和市场准入门槛，完善固定资产投资项目调控管理机制，强化对项目的源头管理。坚持突出重点、区别对待、有保有压，优化投资结构。修订完善工业产业结构调整、工业用地和产业能效指南。大力推进符合产业政策的重点项目，严格控制新上高耗能、高污染和产能过剩行业的项目。加强经济运行预警分析，做好重要生产要素供需监测和协调供应。

　　（二）全面提升自主创新能力

　　积极推进创新型城市建设。争取海尔、海信等国家重点实验室获得批准，加快中科院生物能源与过程研究所、国家深海基地、海洋科学与技术国家实验室建设。重点培育10家企业技术中心。全面落实鼓励企业技术创新的各项政策，推进智能交通、太阳能技术利用、网络家电等关键技术攻关。加强食品药品安全、地方病预防诊治等社会公共领域的技术研发应用。积极争取国家在科研基地、重大科技项目等方面的支持。做好高新区的规划发展。推进与驻青高校和科研院所的合作共建。

　　优化创新环境，激发全社会创新活力。今年市财政安排科技三项经费1亿元，用于重点科技攻关和成果应用转化。进一步完善科技经费使用监管和绩效考核。加快建立规范有效的科技风险投资机制。继续实施知识产权保护战略，支持和帮助企业保护专利成果。建立市级综合性创新创业服务机构和6个区市创新服务平台。加快创新型人才队伍建设，完善人才引进、使用、收入方面的政策，更好地激发创新积极性，营造有利于自主创新的体制环境和文化氛围。

　　（三）加强节能降耗、环境保护和集约用地

　　以能源资源节约为重点，大力发展循环经济。进一步做好节能工作，抓好110家重点用能企业的节能管理，严格落实目标责任和监督考核。设立节能专项资金，促进节能技术改造。实施建筑节能、绿色照明等节能工程。加快开发利用可再生、无污染的清洁能源，推进即墨风力发电、生活垃圾焚烧发电等项目建设，支持秸秆气化、大中型沼气工程和“一池三改”户用沼气发展。加快循环经济和资源节约政府立法。年内实现铬渣全部资源化利用。加强水资源节约和饮用水源地环境保护，完善水资源规划，统一调度水源。争创全国循环经济示范城市。

　　以污染减排、污染整治、强化管理为重点，切实加强环境保护。大力推进清洁生产，对超标排污、使用或排放毒害物质的企业实行强制性清洁生产审核，加大对重点排污企业的监测力度。加强胶州湾水域及近海岸线保护，完成团岛流域和前海一线截污工程，抓好市区北部和五市过城河道的治理。完善北部城区污水管网配套，建设楼山河污水处理厂，改扩建李村河污水处理厂。小涧西垃圾处理厂和麦岛污水处理厂投入使用。加快燃煤锅炉脱硫除尘工程进度。加强放射源、电磁辐射源、固体废物和危险废物监督管理，治理道路、施工扬尘和机动车尾气污染，严格控制大气中可吸入颗粒物的浓度。大力倡导健康、节约、环保的消费模式。

　　坚持集约利用土地，加快建立依法依规用地的长效机制。严格执行国家下达的年度用地指标，严格控制建设用地，严格实行工业用地出让最低价标准，依法规范用地审批程序和土地出让行为，加强土地出让收支管理。规范土地储备制度，提高存量土地使用效率。按照“布局集中、用地集约、产业集聚”的要求，建立和完善开发区土地利用评价指标体系。出台加强地下空间土地使用权管理办法，引导和鼓励利用城市地下空间。

　　（四）推动新农村建设深入发展

　　加快实施城乡统筹发展战略。完善工业反哺农业、城市支持农村的机制，进一步扩大公共财政对农村的覆盖范围，促进农民增收和农村经济社会发展。大力发展现代农业。着力调整农业结构，抓好优质蔬菜、花生和果茶花卉三个100万亩工程建设，推动都市农业、高效渔业和特色畜牧业有新提高。强化农产品安全责任，推行“无公害农产品行动计划”和“出口农产品绿卡行动计划”。推进农业产业化经营，重点支持30家省级以上龙头企业、100个农民专业合作组织、10处农产品产地批发市场和10个高效农业示范园区发展。加强农业技术服务体系建设，实施科技入户工程，积极推广应用农业先进适用技术。不断壮大县域经济。以产业化、集群化、园区化提升工业化水平，加快集群配套向县域延伸的步伐，推动农村工业化特别是平度、莱西工业化实现新发展。完善农村流通设施建设，推进“万村千乡”市场工程。提高县域经济二、三产业比重。

　　加强农村基础设施建设。继续抓好农村水源建设和小型病险水库的除险加固，推进农村除氟改水，完善村村通自来水工程，保障农村饮水安全。加快农村公路、客运站点建设，完成行政村通柏油路500公里。提高农村城镇化建设水平。抓好17个重点镇和100个小康示范村建设，开展1000个村庄的村容村貌整治。做好乡镇污水集中处理和农村垃圾处理系统建设试点工作。

　　大力发展农村社会事业。认真落实新增教育、卫生、文化等事业经费和固定资产投资增量主要用于农村的政策。完善农村义务教育经费保障机制，在县域内统一农村中小学教师工资项目和执行标准，增加农村义务教育阶段的贫困家庭寄宿学生生活费补助和中小学校公用经费，健全城乡教师交流机制。完善新型农村合作医疗制度，参合率达到97%以上。加强农村三级卫生服务网络建设，重建15所农村卫生院，建成100个农村社区卫生服务机构。推进文化共享工程建设，新改扩建1200个村文化活动室。完成124个镇（街道）计生服务站、4000个村计生服务室标准化建设。新型农村社会基本养老保险参保人数增长12%以上，实现被征地农民即征即保。进一步做好库区移民的帮扶工作。

　　深化农村综合改革。积极稳妥地推进县乡财政管理体制改革、农村义务教育改革和乡镇机构改革。继续做好郊区“城中村”新一轮改革。扩大乡镇政务公开、村务公开，完善农村民主政治制度。规范土地承包权流转，做好清理化解村级债务工作，开展农业政策性保险试点。认真组织好第二次农业普查。

　　（五）加强城市建设和奥帆赛筹备工作

　　按照“规划科学化、建设集约化、管理精细化”的原则，推动城市规划建设管理工作上新台阶。

　　提高规划水平。科学合理确定城市空间布局和功能区划，重点抓好增强城市功能、完善公共设施的项目规划。加强滨海公路沿线及周边区域规划控制。做好新一轮市政设施专项规划修编、火车新客站及周边等重点区域的规划控制工作。切实强化规划的权威性，规范审批管理，依法加强执行监督。

　　加快基础设施建设。海底隧道、海湾大桥、即平高速、机场扩建等重点项目按计划进度实施。尽快启动东西快速路三期工程。完成火车站改造主体、杭州支路—鞍山路快速路、长沙路打通等重点工程。加快疏港专用通道及配套基础设施建设。加强建设项目管理，严把工程质量关。做好地铁规划报批工作。提高造林绿化水平，确保通过国家园林城市复检验收。积极申办2009年世界园艺博览会。

　　广泛开展“城市管理年”活动。突出人性化、细节化管理，加强对市容环境、道路交通、景观亮化、重点区域的综合整治，进一步改善城乡面貌和人居环境。开展城管执法专项治理，实施《青岛市城市管理相对集中行政处罚权条例》。建立城市综合管理信息系统，提高城市管理效率和快速反应能力。

　　全力做好奥帆赛筹备工作。高标准完成奥林匹克帆船中心建设，全面整治周边环境秩序，加强对浮山湾海域环境的监控治理。深入实施《人文奥运计划纲要》，营造浓厚的奥运氛围。按照奥运模式和奥运标准，完善工作体制机制，认真做好城市运行、赛事组织、服务保障、安全保卫和迎奥宣传，提高市场开发及运作水平，努力办好2007青岛国际帆船赛。

　　（六）进一步推进改革开放

　　稳步推进重点领域的改革。深化国有企业改革。坚持以产权制度改革为核心，加快国有大企业战略重组。推动资产经营公司的过渡性体制改造和配套改革。进一步完善国有资本经营管理制度，建立国有资本预算收支管理。引导规范大企业经营和投资行为，防范企业经营风险、财务风险，确保国有资产保值增值和品牌安全。加快投融资体制改革。完善重大基础设施项目法人制、招投标制。继续做好企业上市工作。鼓励社会资本投向基础设施、市政公用等领域。稳妥推进事业单位分类改革，完善事业单位聘任制和岗位设置管理。稳步实施社会领域体制改革。适时推进与群众利益密切相关的资源、能源类产品和服务价格改革，保持消费品价格基本稳定。做好“内外资企业所得税税率统一”的有关准备工作。

　　切实提高对外开放质量和水平。不断调整优化出口结构，扩大机电产品、高新技术产品出口规模，争创国家科技兴贸出口创新基地。完善外贸出口预警和贸易摩擦应对服务机制。充分利用国家政策，引导企业增加先进技术、关键设备和重要资源的进口。进一步拓展招商渠道，优化利用外资结构，推进装备制造业、高新技术产业、现代服务业和循环经济等领域的招商引资，积极承接国际服务外包业务。抓好一批重点引资项目。加快出口加工区发展，推进保税区申办保税港区。优化外商投资环境，推进“大通关”和电子口岸建设，做好亲商、安商、抚商工作，增强外商来青投资的信心。推动外事、侨务、对台、国内经济合作交流取得新进展。

　　四、坚持和谐发展，使广大人民群众共享发展成果

　　各位代表！努力解决广大群众最关心、最直接、最现实的利益问题，是构建和谐社会的内在要求，也是政府义不容辞的社会责任。我们将牢固树立以人为本、民生为重的理念，突出公共性和普惠性，切实维护公平正义，促进社会稳定和谐。

　　（一）着力解决民生问题

　　积极促进社会就业。扩大统筹城乡就业试点，完善就业政策和服务体系，推进公共实训基地建设，加强对困难群体的就业援助。全年实现城乡就业20万人，其中农村劳动力转移就业8万人。健全收入分配制度，研究建立企业工资与效益同步增长机制，完善工资指导线、最低工资标准和最低生活保障线适时调整的机制，提高居民特别是低收入者收入水平。健全农民工工资支付保障和监控制度。

　　完善社会保障体系。抓好社会保险扩面征缴，尽快使养老保险、医疗保险、工伤保险覆盖所有企业，逐步做实企业职工基本养老保险个人账户。继续增加企业退休人员养老金。建立城镇居民基本医疗保险制度。扩大医疗救助对象和救助病种范围，提高临时救助标准。继续做好外来务工人员社会保障工作。改扩建市救助管理站，建立市孤残儿童养护中心。大力发展慈善和福利事业。加强对各类社保基金、公益金、公积金的监督管理，提高使用效益。

　　加强居民住房建设。以健全保障体系、增加住房供应、稳定住房价格为重点，加大中低价位、中小套型住房建设力度。建设普通商品住房、经济适用住房、租赁住房、廉租住房342万平方米，比上年增加20%。全年工作目标是：开工旧城区改造项目9个，城中村改造项目9个；增加廉租住房保障户数，新增租赁住房保障方式；新建住房销售价格增幅低于上年，普通商品住房销售价格低于上年；经济适用住房销售价格保持稳定。

　　改善生活环境质量。优先发展公共交通，科学调整站点设置，新增公交线路，年内置换更新公交车400辆。完善停车场规划，建设舞阳路、河马石二期等7处公交停车场和八大峡等2处旅游停车场。提高城区供热能力和供热质量，新增供热面积300万平方米。建立对政府特许经营公用行业公开公正的审计、考核评价制度。

　　完成市政府今年重点办好的居民住房建设、食品安全体系建设等10件实事。

　　（二）加快发展社会事业

　　坚持教育优先发展。贯彻落实《义务教育法》，促进城乡义务教育均衡发展，今年免除城镇义务教育阶段学生的杂费。加强素质教育，稳步推进中考制度改革。支持高等教育发展，大力发展职业教育，引导民办教育健康发展。完善弱势群体就学保障机制，扩大贫困家庭学生就学资助覆盖面。规范改制学校体制。在全市中小学校（托幼园所）推行校方责任险，防范和妥善化解学校安全事故风险。进一步规范教育收费项目和标准，坚决制止乱收费。

　　加快发展卫生事业。围绕建立覆盖城乡居民的基本卫生保健制度，强化政府责任，深化医疗卫生体制改革，提高医疗卫生服务水平。加快发展社区医疗，建设12所“六位一体”的社区卫生服务示范中心。加强公共卫生体系建设，做好重大疫情和传染病防治工作。严格医疗机构监管，确保医疗安全。推进国医堂、中心医院病房楼、中心血站业务楼改造等项目建设。加强学科建设，加快人才培养和引进。开展对贫困家庭患白血病、先天性心脏病等儿童的医疗救助。

　　大力推进文化建设。抓好青岛大剧院、特色文化街区等重点文化基地建设。培育“帆船之都”等城市品牌。办好奥林匹克文化节、青岛音乐节等重大活动。着力发展影视、动漫、演艺等文化产业，推动其多元化发展。加强文化遗产保护。

　　促进社会事业全面发展。加快市体育馆等场馆及配套设施规划建设。广泛开展“与奥运同行”为主题的全民健身活动，提高竞技体育水平，争取在第6届全国城运会上取得优异成绩。加强国防动员和国防教育，推进民兵预备役建设，高标准做好双拥工作，力争第六次获得“全国双拥模范城”。稳定低生育水平，提高出生人口素质，完善流动人口管理服务体系，统筹解决人口问题。高度重视老龄事业发展，加快推进养老服务社会化，逐步形成以社会供养为主、家庭供养为辅的养老模式。继续实施养老服务“双千”计划，加快市区老年公寓建设。新建乡镇中心敬老院19所，改扩建30所，实现镇镇有中心敬老院。支持工会、共青团、妇联等人民团体发挥桥梁和纽带作用。认真落实妇女儿童发展纲要，加强未成年人权益保护，推动残疾人事业发展。促进广播电视、新闻出版、社会科学、民族宗教、档案史志、气象、地震、红十字、人防、仲裁等工作全面进步。

　　（三）全面推进文明创建工作

　　围绕建设社会主义核心价值体系，深入贯彻《公民道德建设实施纲要》，广泛开展理想信念教育和社会主义荣辱观教育，大力弘扬积极向上的主旋律，凝聚力量，增强信心，营造健康思想舆论氛围和知荣辱、讲正气、促和谐的社会风尚。加强政务诚信、商务诚信、社会诚信建设，增强全社会诚实守信意识。

　　深入开展文明和谐创建活动。增强社会成员和主体的社会责任，积极创建和谐家庭、和谐社区、和谐村镇、和谐企业，不断提高市民文明素质和城乡文明和谐程度。广泛开展“同迎奥帆赛、共建文明城”、文明交通“三让”、志愿者服务、无偿献血、慈善关爱等活动。完善文明创建考评细则，推动创建工作规范化，办好图书文化节暨全民“读书月”活动。

　　（四）切实加强社会管理

　　推进“平安青岛”建设。按照构建社会主义和谐社会的要求，加强社会治安综合治理。以化解社会矛盾为主线，完善“大调解”工作机制，加强司法保障，提高司法机关社会管理能力。健全信访工作责任制，建立全市信访信息系统，依法规范信访秩序。深化“十百千”基层创安和平安行业规范化建设活动，依法严厉打击严重刑事犯罪，完善社会治安防控体系建设，增强人民群众的安全感。妥善解决刑释解教人员安置帮教和青少年违法犯罪预防等领域的突出问题。

　　加快城市应急指挥平台建设，健全应急管理体制，提高应对突发事件的能力。严格落实安全生产责任制，加强重点行业和重点领域的安全监管，建设黄岛石化区安全生产监控系统，实施八水河道路交通安全隐患整改方案，坚决遏制重特大事故发生。继续整顿和规范食品药品市场秩序，保障人民群众饮食用药安全，努力创建全国食品最安全城市。

　　加强社区建设。进一步落实社区工作有关政策，健全社区民主自治制度。完善新型社区管理服务机制，逐步建立社会福利服务、社区互助服务、市场有偿服务相结合的社区服务网络体系。发挥驻区单位、组织、机构在社区建设中的作用。深入推进农村社区建设，完善村（居）民自治，维护农村和谐稳定。

　　五、坚持依法行政，切实提高政府服务水平

　　当前，青岛正处于改革发展稳定的关键时期。我们将坚持全心全意为人民服务的宗旨，认真履行政府工作职责，努力为人民群众提供更好的服务，为经济社会发展营造更好的环境。

　　坚持科学理政，加快职能转变。认真贯彻《公务员法》，深入开展创建高绩效机关、做人民满意公务员工作，进一步提高公务员素质。努力学习现代经济知识、科技知识、社会管理知识和法律知识，全面提高驾驭市场经济的能力、推进自主创新的组织领导能力、管理社会的能力和依法办事的能力。深化行政管理体制改革，推进体制、机制、制度和管理创新。在认真抓好经济调节和市场监管的同时，更加注重履行社会管理和公共服务职能，逐步形成惠及全民的基本公共服务体系。加强电子政务建设，推进政府管理服务信息化。

　　坚持民主施政，建立决策、执行、监督相协调的政务机制。全面贯彻执行《监督法》，自觉接受人大及其常委会的法律监督和工作监督，接受政协的民主监督以及社会舆论监督，支持监察、审计部门依法独立开展工作。完善落实特约人员工作制度，充分发挥各民主党派、工商联和无党派人士的民主监督作用。严格政府各类资金的审批和监管，提高资金使用效益，防止违法违规使用。完善重大决策专家咨询、社会公示和听证制度，健全决策反馈纠偏机制和决策责任追究制度。建立规范高效的执行机制，严格落实行政问责制，认真查处各种不作为、乱作为的行为。实行政府内部考核与公众评议、专家评价相结合的绩效评估办法。组织市民开展为青岛发展献计策活动。深化政务公开，保障人民群众的知情权、参与权、表达权和监督权。

　　坚持依法行政，加快建设法治政府。严格依照法定权限和程序行使权力、履行职责，确保权力公开、公平、公正运行。深入实施《行政许可法》，进一步规范现有行政许可，减少非行政许可审批项目，加快推进行政审批电子管理及监察系统建设。强化行政执法责任追究，健全政府重大决策及相关文件的法律审查制度。

　　坚持从严治政，进一步加强作风建设和廉政建设。全面加强思想作风、学风、工作作风、领导作风和干部生活作风建设，弘扬新风正气，抵制歪风邪气，着力解决突出问题。坚持求真务实的工作作风，坚决反对形式主义、官僚主义和弄虚作假。坚决反对铺张浪费和大手大脚，自觉抵制拜金主义、享乐主义和奢靡之风。规范公务接待行为。贯彻标本兼治、综合治理、惩防并举、注重预防的方针，认真落实市委《关于进一步加强党风廉政建设和反腐败工作的意见》，深入推进惩治和预防腐败体系建设，拓展从源头上防治腐败的工作领域。以工程建设项目招投标、土地使用权出让、企业国有产权交易、政府采购、规划审批等领域为重点，深化改革、完善制度、强化监督，努力形成按制度办事、靠制度管人的机制。政府领导干部要自觉遵守和严格执行政治纪律、工作纪律、组织纪律和廉政纪律，严格落实“一岗双责”制度。认真抓好各级各部门一把手的教育、监督和廉洁自律，坚决惩治官商勾结、商业贿赂等腐败行为，严厉查处侵害群众利益的案件，以更坚决的态度、更有力的措施、更扎实的工作，把党风廉政建设和反腐败斗争推向深入。

　　各位代表！全面做好今年的各项工作，任务艰巨，意义重大。让我们更加紧密地团结在以胡锦涛同志为总书记的党中央周围，高举邓小平理论和“三个代表”重要思想伟大旗帜，坚持以科学发展观统领全局，在中共青岛市委的正确领导下，团结依靠全市人民，振奋精神，坚定信心，奋发进取，以崭新的风貌、优异的成绩，迎接党的十七大、省九次党代会和市十次党代会的胜利召开！
